# Environmental Epigenetics: Crossroad between Public Health, Lifestyle, and Cancer Prevention

**DOI:** 10.1155/2015/587983

**Published:** 2015-08-03

**Authors:** Massimo Romani, Maria Pia Pistillo, Barbara Banelli

**Affiliations:** Laboratory of Tumor Epigenetics, IRCCS AOU San Martino-IST, Largo Rosanna Benzi 10, 16132 Genova, Italy

## Abstract

Epigenetics provides the key to transform the genetic information into phenotype and because of its reversibility it is considered an ideal target for therapeutic interventions. This paper reviews the basic mechanisms of epigenetic control: DNA methylation, histone modifications, chromatin remodeling, and ncRNA expression and their role in disease development. We describe also the influence of the environment, lifestyle, nutritional habits, and the psychological influence on epigenetic marks and how these factors are related to cancer and other diseases development. Finally we discuss the potential use of natural epigenetic modifiers in the chemoprevention of cancer to link together public health, environment, and lifestyle.

## 1. Introduction

The complete sequence of the human genome was released at the beginning of the XXI century and can be considered as the master library where all the genetic information is stored. All this information, to be used, must be properly read and interpreted. Even a well-known text like the Hamlet soliloquy “to be, or not to be…” would be hard to understand without word interruptions and punctuation. Indeed, the genetic code requires another code on top of it (from the Greek *επι*) that, like the annotations on the side of a book, enables the comprehension of the text [1]. The term “epigenetic landscape” was coined in 1939 by CH Waddington, before DNA was recognized as the molecule of inheritance, to describe the mechanisms of transition of the cells from the totipotent to the differentiated condition. In practice, epigenetics provides the tools to translate the information (genotype) into function (phenotype) [2]. Thus, if the sequence of the DNA stores all the data necessary to build a living cell or organism, epigenetic, like the operating system of a computer, decodes the information and determine when, how, and where a given set of instructions must be used.

Although epigenetic research has been traditionally focused on developmental and cancer-related alterations [3–5], the effect of the environment and of dietary factors on the epigenetic asset of the live beings is now being increasingly appreciated and the role of epigenetics in nontumor pathologic conditions is actively investigated [6, 7]. Epigenetic modulation occurs during the entire lifespan from conception to adulthood. Maternal diet, alcohol consumption, and smoke habits can all influence the epigenetic landscape, as well as later in life the exposure to environmental chemicals can disrupt the epigenetic programming [8, 9] beside increasing the cancer risk.

Epigenetic mechanisms are involved in drug resistance [10–12] and are responsible, at least in part, for the interindividual variation of the response to drugs [10]. In this respect, a particularly promising avenue for epigenetics is the development of new and effective therapies that could overcome drug resistance. The information contained in the DNA needs to be timely used; in this respect epigenetic modifications, because of their reversibility and rapid change, confer phenotypic plasticity in response to environmental or internal stimuli. From a therapeutic point of view, the reversibility of the epigenetic variations makes them ideal drug targets [11]; indeed in some experimental models drug resistance was found to be reversible and mediated by epigenetic mechanisms [12]. Along this line some reports have shown the clinical utility of drug rechallenge and the possibility to resensitize the patients to first line chemotherapeutic agents intervening on the epigenome [13–18]. Only four drugs are currently utilized as single agents or in combination for the therapy of Myelodysplastic Syndrome and Cutaneous T Cell Leukemia and other hematological disorders (Table 1). However, at the end of 2014, 195 open trials based on or including epigenetic drugs were listed in the database of the clinical trials of the NIH (https://clinicaltrials.gov/). Noticeably, several of these ongoing trials are not aimed at treating cancer diseases (Table 2) indicating that the potential therapeutic use of  “epigenetic drugs” is extending beyond the boundaries of cancer.

In this review we will discuss the interaction between the environment and the epigenome and how natural and synthetic molecules that modulate epigenetic factors can have preventive properties against cancer.

## 2. Mechanistic Aspects of Epigenetic Regulation

Epigenetic inheritance occurs through four basic layers deeply interconnected:

1. DNA methylation and hydroxymethylation,
2. histone modification,
3. chromatin remodeling,
4. ncRNA.

Epigenetic modifications are controlled by a set of enzymes whose functions can be summarized as follows: the “writers” are the enzymes that modify their target by adding residues (i.e., methyl groups to DNA or histones); the “erasers” remove the added residues and the “readers” are the proteins that recognize and bind to the modified targets and act as intermediate for subsequent protein-protein interactions (Table 3).

The number of epigenetic modifier proteins is steadily increasing: in 2009 they were “only” 91 and in 2012 more than 109 different proteins were identified.

### 2.1. DNA Methylation and Hydroxymethylation

DNA methylation is the most widely studied epigenetic alteration and was the first one to be linked to cancer [19]. The only biologically relevant C-methylation occurs at CpG doublets and is mediated by DNA methyltransferases (DNMTs) that catalyze the addition of a methyl group at C-5 of the cytosine. It is generally accepted that only DNMT1, DNMT3a, and DNMT3b are capable of C-methylation and that DNMT1 is responsible, mainly but not exclusively, of the maintenance of DNA methylation throughout development and cell life, thus preserving genomic integrity [20]. DNMT3a and DNMT3b are generally accepted as* de novo* DNMT for setting DNA methylation patterns. However,* de novo* and maintenance DNMTs are not tightly compartmentalized. The bulk of DNA methylation is carried out by DNMT1 which is also capable of* de novo* methylation. On the other hand, DNMT3a and DNMT3b can methylate the CpGs missed by DNMT1 at the replication fork thus serving as “maintenance DNMTs.” The human genome contains approximately 3 × 10^7^ CpGs and although methylation at single doublets may, in principle, have functional consequences [21, 22], the biologically relevant DNA methylation is occurring at CpG cluster (CpG islands) [23, 24]. These clusters can be localized at the gene promoter and their methylation is inversely associated with transcription. CpG islands can be also intergenic or intragenic; these clusters are generally hypermethylated to prevent spurious initiation particularly at internal promoters. An example of inappropriate transcriptional activation is that of* ΔN-p73*, oncogenic and antiapoptotic variants of the* p73* gene from an internal promoter kept silenced by methylation of a small CpG island [25–27].

DNA methylation is deeply altered in cancer cells that present diffuse hypomethylation along with focal hypermethylation of selected genes or regions of the genome. The general idea is that hypomethylation contributes to genomic instability while hypermethylation inactivates tumor suppressor genes. However, the picture is likely much more complex and methylation changes outside promoter regions may have unexpected effects on gene expression [28, 29]. Aberrant DNA methylation is not restricted to cancer and is present also in disorders of imprinting like the Beckwith-Wiedemann syndrome [30], the Prader-Willi syndrome, the ICF syndrome [31], and other neurodevelopmental disorders [32].

5-Hydroxymethylcytosine (5-hmC) is considered as the “sixth base” of the genome, with 5-methylcytosine (5-mC) being the “fifth,” and is an intermediate in the removal of methyl groups from cytosine by the TET 1 enzymes. 5-hmC has the opposite function of 5-mC and is a transcriptional activator [33, 34]. It is not clear if the mechanism through which 5-hmC activates transcription is the removal of the methyl group with the consequent displacement of the MDB or if TET proteins act as “readers” preventing the binding of DNMT.

### 2.2. Histone Modifications

DNA is tightly compacted around an octamer of histones forming a structure named “nucleosome” which is the basic unit of the chromatin and includes 146 bp of DNA wrapped around a disk-like structure composed of two molecules each of H2A, H2B, H3, and H4. A 80 bp linker DNA and a fifth histone (H1) separate adjacent nucleosomes. The electron microscopy appearance of the chromatin is that of a “beads-on-a-string” whose packaging is determined by the histones.

Histones for many years were considered simply as structural proteins whose function was to assemble DNA into chromosomes. Only in the 1960s Allfrey at al. [35, 36] showed that histones undergo postsynthetic modifications that are related to the control of transcription. Because of his pioneeristic work, Allfrey must be considered one of the fathers of epigenetics. Beside the five major histones, several variants with unique distribution patterns and functions not exerted by the “classic” histones have been described [36, 37]. The N-terminal tail of histones is subjected to various types of modification including acetylation, methylation, phosphorylation, ubiquitination, ADP-ribosylation, and biotinylation. The effects on transcription of some of these modifications are reported in Table 4. Histone modifications occur at specific sites and in various combinations and, along with the discovery of specific functions of histone variants, generated the hypothesis of the “Histone Code” that postulates that distinct modifications, alone, or in combination or sequentially can be read by effector proteins to bring about downstream events [38].

Initially, the transcriptional control mechanism of histone modifications was considered to be primarily “mechanic.” The transfer of acetyl groups mediated by Histone Acetyltransferases (HAT) to the lysines of histone tails neutralizes the positive charge of the AA and results in the weakening of the interaction with DNA [39, 40] and in a “relaxed” chromatin conformation. Deacetylation of histones by histone deacethylases (HDAC) restores the positive charge of lysines and the “closed” chromatin conformation. It is believed that the opening of the chromatin is a key step for the recruitment of the transcription machinery; thus, HAT and HDAC are transcriptional activators and silencers, respectively. Similarly, the phosphorylation of serine, threonine, and tyrosine mediated by kinases and phosphatases changes the net charge of histones contributing to the changes of the chromatin structure.

Also the methylation of histones occurring at lysine and arginine was discovered by Allfrey et al. in 1964 [40]. Differently from acetylation and phosphorylation, methylation does not change the protein charge and does not modify the interaction between DNA and histones. For many years histone methylation was considered an irreversible modification and only in 2004 the first histone demethylase (KDM), the LSD1 (KDM1) amine oxidase, was identified [41]. Since then many other KDM genes were identified and they represent a complex and expanding family involved in many aspects of cell physiology and pathology [42–44]. Several KDM genes act as oncogenes or antioncogenes, are involved in anticancer drug response, and have been proposed as therapeutic targets [12, 43, 45, 46]. The effect of methylation of histone tails on transcription is that of recruiting effector proteins with promoting or inhibiting properties.

### 2.3. Chromatin Remodeling

The tight packaging of chromatin prevents the binding of transcription factors and RNA polymerases. Chromatin remodeling can be obtained not only by histone acetylation/deacetylation but also through ATP-dependent protein complex formation, histone modification by polycomb proteins (PcG), and by interaction of noncoding RNA (ncRNA).

Nucleosomes can be repositioned by the SWI/SNF ATP-dependent complex through the formation of a DNA loop that moves repositioning the nucleosome and reduces the distance between adjacent nucleosomes [47, 48]. This process may activate transcription in regions where nucleosomes are relaxed and more distant and can silence the regions where chromatin is more compact.

PcG proteins repress transcription compacting the chromatin through DNA methylation and multiple histone modifications following a multistep protein recruitment that prevents mRNA elongation [49, 50].

ncRNA can influence transcription by targeting PcG proteins to specific sets of genes [51]. The interaction between ncRNA and PcG is particularly important in chromosome X inactivation. A ncRNA (*Xist*) is expressed from the noncoding X chromosome, recruits a PcG complex denoted as PRC2, and initiates silencing by binding the X chromosome [52].

### 2.4. ncRNA

ncRNA are a class of RNA that are not translated into proteins, whose function is that of controlling the processing and function of other RNAs thus intervening in complex pathways and cell mechanisms. The principal ncRNA and their functions are reported in Table 5.

In general ncRNAs interfere with the functionality of other RNAs through a mechanism called RNA interference (RNAi). RNAi regulates gene expression in a sequence-specific way without altering the target sequence; accordingly this mechanism is considered epigenetic. The role of micro-RNAs as epigenetic controllers is well recognized and this class of ncRNA is the most studied in this respect. miRNAs inactivate transcription by base pairing of nucleotides 2–8 (the “seed” region of miRNA) and the 3′UTR of the mRNA. This leads to premature degradation or to the stop of translation through the formation of the “silencing complex” [53–55]. miRNAs are involved in a variety of biological processes including cell growth and differentiation, apoptosis, maintenance of cell identity and they are deregulated in cancer and other diseases [56]. A miRNA can have multiple targets and a gene can be targeted by many miRNAs; thus, the regulatory pathway determined by these molecules can be extraordinary complex.

Interestingly, miRNAs expression can be modulated by DNA methylation and histone modifications and they can modulate the DNA methylation machinery leading to a “loop” of epigenetic regulation [57–59].

Importantly and interestingly, exogenous miRNA sequences may enter the cells from the outside and exert their biological effect [60] demonstrating that the environment can have profound effects on the epigenome.

## 3. Influence of the Environment on Epigenetic Marks

Nucleated somatic cells of the human body contain approximately 3 billion bp of DNA that, in length, correspond to a filament of roughly 3 meters. Although tightly packaged in chromatin and hidden in the nucleus, DNA is exposed to a variety of agents that may influence epigenetic regulation. DNA methylation is considered a stable modification; however, it is sensitive to multiple agents including those that reduce the bioavailability of S-adenosylmethionine, the major methyl donor involved in DNA methylation [61]. Intuitively, the highly dynamic histone modifications are very sensitive to the environmental changes since each of the many enzymes involved in these processes is a potential target of epigenetic regulation. Moreover, drugs and dietary compounds and the exposure to contaminants that pass the placenta can produce subtle alterations of the developmental pattern of the fetus [62, 63]. According to the “developmental origin of adult health and disease” hypothesis [64, 65], exposure to chemicals or natural bioactive products during pregnancy may have long-term effects on health also through epigenetic mechanisms.

### 3.1. Epigenetic and Lifestyle

The environmental agents that can interfere with DNA methylation are widespread and depend on lifestyles. Smoking, alcohol consumption, UV light exposure, or factors linked to oxidative stress are some of the most common and important lifestyle aspects that may alter the DNA methylation profile [66].

The effects of smoke on DNA methylation have been extensively studied but the results obtained are contradictory and no consensus has yet been reached. Indeed, if Benzo[a]pyrene has no effect on the methylation of the DNA sequences known to be involved in lung cancer [67], smoke condensate containing the full spectrum of carcinogenic substances found in cigarettes modifies the methylation patterns of tumor suppressor genes involved in the early stages of lung carcinogenesis [68, 69].

Experimental studies failed to demonstrate the direct carcinogenic activity of alcohol; however, the causal relation between alcohol intake and cancer has been established several years ago [70]. In a Dutch study on diet and cancer it has been demonstrated that the methylation pattern of tumor suppressor and DNA repair genes was altered in colorectal cancer patients with low folate/high alcohol intake [71]. However, a subsequent study failed to show the significant association between folate intake, alcohol and methylation of* MLH1*, a gene hypermethylated early during colon carcinogenesis [72]. It must be pointed out that these studies were conducted using qualitative methylation analysis techniques that may not be sufficiently accurate to disclose subtle, but clinically relevant differences of methylation. In this respect it has been demonstrated that quantitative methylation analysis offers significant advantages over purely qualitative techniques and enables to identify methylation cut-off values that define disease-specific methylation patterns [73–78].

Alcohol-related epigenetic changes influence neuronal growth and development and affect memory and learning possibly modifying the methylation status of many genes. In particular, a genome-wide methylation analysis, followed by stringent validation by quantitative methodologies, showed remarkable differences in 84 genes involved in brain metabolism and differentiation [79]. This study indicated that exposure to alcohol of early mouse embryo changed the methylation pattern and the expression of several genes and these alterations were put in relation with neural tube defects. Interestingly, the human homologues of many of the genes epigenetically modified by acute alcohol administration in mice are involved in neurologic pathologies like Alzheimer disease (APP), ALS-Parkinson (TRPM7), myotonic dystrophy (DMPK), Angelman syndrome (UBE3A), and others.

### 3.2. Endocrine Disrupting Chemicals in the Environment and Epigenetics

A particularly serious and often poorly considered effect of the accumulation of chemicals in the environment is the alteration of the endocrine signaling pathways by chemicals (denoted by “Endocrine Disrupting Chemicals” (EDCs)) that mimic the action of the natural molecules [80].

Considering that these chemicals are utilized since the 1940s and that some of them, like biphenyls, are extremely resistant to degradation while the metabolites of others, like DDT, are biologically very active, it is not surprising that they are considered to constitute a health hazard.

The effect of EDC has been considered to be transgenerational and it is believed that these molecules could affect also the offspring through epigenetic mechanisms [81, 82].

Epigenetic inheritance has been advocated for many pathologic conditions and many experimental evidences have been accumulated in support of this hypothesis [83–86]. The concept of transgenerational epigenetic inheritance is very attractive and it has been demonstrated for plants and lower organisms like nematodes. However, recent surveys of the literature have raised several questions in particular on the role of the environment in epigenetic inheritance and on its extent in mammals [87, 88].

One of the most common EDCs found in the environment is Bisphenol A (BPA) a compound used in the manufacture of polycarbonate and epoxy resin. The most common mode of assumption of BPA in humans is through canned food since epoxy resin coating food cans and releases BPA. It has been estimated that the intake from food of this chemical is approximately 7 *µ*g/person/day. BPA and its derivatives can be easily detected in bodily fluids at bioactive concentrations [80]. Several reports describe the effect of BPA on epigenetic programming and demonstrate that this chemical can alter the expression of selected genes through histone methylation, DNA methylation changes, and miRNA expression. The exposure to BPA, in developmental* in vivo* models, increases the susceptibility to prostate cancer and it is likely involved in breast carcinogenesis [89–91]. In another experimental model, the continuous exposure of mice before mating and during gestation and lactation produced hypomethylation of the DNA, obesity, and diabetes and increased tumorigenicity in the offspring [92]. Interestingly, the epigenetic alterations induced by BPA could be counteracted by dietary supplementation with methyl donors [92].

Diethylstilbestrol (DES), a synthetic estrogen antagonist widely utilized between 1938 and 1971, induces vaginal cancer in the offspring of the treated mothers [93]. Persistent epigenetic changes that can be passed to the next generation can be induced by DES treatment [94, 95]. Among the genes whose expression and/or methylation is altered by* in utero* exposure to DES, two are particularly relevant for cancer development: the* EZH2* histone methyltransferase involved in breast cancer, in glioblastoma, and in other tumors [96, 97] and the* HOXA10* gene, involved in cell stemness and in glioblastoma [75, 98].

Beside DTT, other insecticides and pesticides, like Methoxychlor, Vinclozolin, and the widely used Permethrin, are widespread environmental contaminants and EDC; some of them have various effects on the epigenome since they can alter DNA methylation as well as the level of expression of the DNA methyltransferase* DNMT3B* [82, 99–101]. Several genes, including the paternally imprinted* H19*,* Gtl2*, and* Meg3* and the maternally imprinted* Peg1*,* Peg3*, and* Snrp* genes, are affected through altered CpG methylation levels in exposed animals. Interestingly, the effect persisted through three generations although diminished from F1 to F3 [102].

### 3.3. Nutrition and Epigenetics

The effect of the dietary habits on health and disease prevention has been the subject of many investigations and a proper and balanced diet is one of the key points of a “good lifestyle.” Through food we are exposed daily to many toxic substances like, for example, BPA. At the same time certain components of the diet can modify the epigenetic pattern through natural bioactive components that can act on DNA methylation or histone modification [103–106] or, as in the case of exogenous miRNA, that can be direct epigenetic actors [60]. In a recent survey on the effects of dietary compounds on the basic mechanisms of epigenetics, it has been recognized that many vegetable components have detectable activities on HAT in human subjects [107, 108].

**Figure 1:**
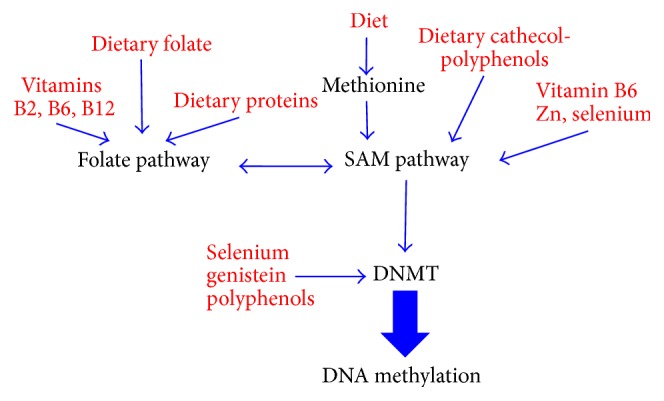
One-carbon metabolism and DNA methylation schematic representation of the interaction of dietary components on folate and SAM pathways and their effect on DNA methylation.

Several nutrients in the diet have a key role in the methylation of all biological substrates and can influence DNA methylation either by changing the availability of methyl donors or by modulation of the DNMTs activity (Figure 1) [109]. In particular, folic acid and Vitamins B6 and B12 are essential for the one-carbon metabolism and their insufficient dietary intake (or an excess) can alter the availability of S-adenosylmethionine, the methyl donor for DNA methylation. Folic acid prevents neural tube defects and in 1998 has led the FDA to require that certain foods and dietary supplements are enriched with folates. The* in vivo* effect of folic acid administration on DNA methylation has been well documented and intake or deprivation of folic acid in colon cancer and healthy individuals resulted in increase or decrease of methylation in colon mucosa and lymphocytes, respectively [110–113]. However, the impact of exogenous folate administration and of the modulation of DNA methylation in humans has not been fully determined as it will be discussed in more details in another section of this review. In particular, it is not clear how dosages and the length of the treatment affect DNA methylation and if there is a time-window,* in utero*, when treatment could significantly overcome nutritional deficiencies. As discussed earlier, the maternal exposure to epigenetic modifiers can affect the transmission of epigenetically controlled traits. In principle, the same effect can result from the administration with the diet of nutrients affecting the epigenome. Indeed, in mice, the addition of folic acid and choline, both involved in one-carbon metabolism, can increase the methylation of* IGF2* [114], an imprinted gene whose loss of imprinting is involved in colon cancer [115], and can alter histone modification possibly through the increase of histone methyltransferase expression [116]. In humans, understanding the effect on the offspring's epigenome of folic acid administration to pregnant women is complicated by the widespread utilization of folic acid as food supplement.

In animals, the effect of maternal diet on epigenetic marks has been widely studied and the results showed the complexity of the interplay between many different components [109].

In humans the impact of the diet on developmental reprogramming has never been formally proven; however, the effect of famine on the imprinting of the* IGF2* locus has been evaluated in 60 individuals of the “Hunger Winter Families Study” [117] conceived in Holland and in their siblings and in matched controls. The results of this study showed that the methylation level of* IGF2* was significantly lower in the individuals periconceptionally exposed to famine compared to the controls [118] supporting the hypothesis that epigenetic changes occurred in the early-life can be maintained during adulthood. However, the phenotypic consequences of the periconceptional exposure to epigenetic-modifying conditions are not known.

Folic acid and vitamins B2, B6, and B12 are not the only dietary components acting on the epigenome. Polyphenols, found in green tea and vegetables, are a group of natural compounds that do not modify the availability of methyl groups but interfere with the activity of DNMTs, HATs, and HDACs [119, 120]. Polyphenols have gained a particular interest when it was shown that they could revert malignancy-associated epigenetic alterations in cell lines [121–123] and that they could modulate DNA methylation in humans [124, 125].

Phytoestrogens are another group of naturally occurring molecules found predominantly in soybeans that inhibit DNMT1, DNMT3a, and DNMT3b and HDAC that were hypothesized to reduce the risk of hormone-related tumors [126–128].

Selenium is an essential component of the diet that can interfere with the epigenome by inhibiting DNMT and HDAC (Figure 1). Importantly, selenium can reactivate the expression of genes involved in the response to oxidative stress and to protection from carcinogen [129, 130].

The effects of nutrition at the early stages of gestation and in the early postnatal life are part of the complex field of disease chemoprevention that will be discussed in another part of this review.

### 3.4. Psycho-Epigenetics

The early-life experiences are considered the building blocks for adulthood and their importance is well recognized by psychologists. Alteration of the methylation levels of neuronal glucocorticoid receptors in hippocampus of suicide victims was put in relation with childhood abuse and similar results were obtained in rats exposed to stress [131, 132].

Although all these data support the concept that epigenetic mechanisms are regulated, by dietary and environmental factors, and may have an influence on health and behavior, the functional consequences of this type of control are largely incomplete and a task for future research will be to link, at the genome-wide and population level, epigenetic changes, expression of the genes, and the resulting phenotype.

## 4. Role of Epigenetics in Chemoprevention

The possibility of modulating the epigenome through the diet opens the possibility of utilizing bioactive molecules for the prevention of diseases characterized by the alteration of the epigenetic asset. Intuitively, the utilization of synthetic or natural molecules with hypothetic chemopreventive properties was pioneered mainly for cancer where the effect of demethylating the genome is beneficial [133]. Indeed, along with the generalized hypomethylation that, supposedly, activates genes with oncogenic properties, cancer cells have a distinct hypermethylation profile that frequently involves genes with antioncogenic properties. The rationale for a hypomethylating therapy is to reactivate these dormant genes to counteract the effect of the active oncogenes. The basic concept of chemoprevention is to prevent the initiation or progression of premalignant lesions through the administration of synthetic compounds or through food additives. Ideally, chemoprevention should be focused toward individual at risk either familiar or because they are exposed to carcinogen. Epigenetic chemoprevention is particularly attractive because epigenetic alterations are reversible and are an early event in cancer development [134, 135].

The major drawback of epigenetic therapies is the lack of specificity and the global hypomethylation achieved by DNMT inhibitors might be highly detrimental. Indeed, repetitive elements and cryptic internal promoter are normally kept hypermethylated but can be reactivated by epigenetic drugs. This leads to genomic instability and to the illegitimate transcription of genes that, at a certain stage of development, should not be functional [136–139].

Thus, DNMT inhibitors, along with recognized anticancer activity, can promote oncogenic transformation that could be counteracted only with more specific drugs or by combination therapies targeting other epigenetic determinants and pathways [140].

### 4.1. Synthetic Drugs

Synthetic inhibitors of DNMT and HDAC are being utilized mainly as therapeutic drugs rather than as chemopreventive agents in hematopoietic tumors [141–143]. These molecules inhibit DNA methylases by incorporating into DNA (5-Azacytidine and 5-Aza-2-deoxycytidine) or inhibit histone deacethylase (SAHA and TSA).

To be active, nucleoside analogues require their incorporation into DNA; for this reason, they are more effective on rapidly proliferating cells rather than on quiescent or nearly quiescent cells.

Inhibitors of HDAC and of DNA synergize together and are being utilized in combination in clinical trials. TSA and SAHA cannot reactivate hypermethylated genes unless a minimal demethylation is achieved by DNMT inhibition [144].

### 4.2. Natural Bioactive Food Components

We have previously seen that some diet components (folates, Vitamins B2, B6, and B12) are essential components of the one-carbon metabolisms and are involved in global DNA methylation. The cancer preventive effects of folates are, likely, tissue specific and age dependent. In rats, maternal supplementation of folic acid increases the methylation in the colon of the offspring and reduced by 64% the risk of colorectal cancer [145]. However, the same treatment reduced the overall methylation in mammary glands and doubled the risk of mammary carcinoma [146]. Folic acid can increase overall methylation in liver; however, this effect seems to be age-related occurring in mice older than 18 months but not in those below 4 months of age [147]. Trials in human subjects were contradictory. In a randomized trial, folic acid supplementation did not change the recurrence rate of colorectal adenoma in patients with a previous history of adenoma [148]. In another study conducted on healthy subjects, folic acid administration for 3 years resulted in the persistent methylation of cancer-related genes in the colon [149].

Folates and related drugs interfere with global methylation, and other molecules present some specificity in their action. Polyphenols, like epigallocatechin-3-gallate (EGCG), curcumin, and resveratrol are three molecules widely distributed in vegetables and other dietary components that are being studied for their anticancer properties [120]. Polyphenols exert their activity through several mechanisms: carcinogen detoxification, DNA repair, cell cycle progression, activation of differentiation, and epigenetic modulation. EGCG is active against many types of cancer cells [150], demethylates and reactivates several genes involved in cancer like* p16*,* RARβ*,* MGMT*,* hMLH1,* and* GSTP1*. Although EGCG seems to be a competitive inhibitor of DNMT1 [123], it can also have apparent demethylating activities or be inactive in some biological systems [151, 152].

Genistein, a phytoestrogen extracted from soybean, can interfere with multiple pathways through hormone receptors and has the same activities of EGCG on* p16*,* RARβ,* and* MGMT* [153]. Many* in vitro* studies have documented the antiproliferative and anticancer activities of genistein particularly, but not exclusively, in prostate and breast cancer [154, 155].* In vivo* model studies have shown that genistein can modify methylation levels in the prostate of mice [156] and that, overall, epigenetic changes could be detected by genistein treatment; however, the results on the anticancer activity of this compound were contradictory. Genistein seems to have protective effects against induced carcinogenesis [157] but it was also observed that genistein could even stimulate tumor growth through hormone receptors [153]. Moreover, the plasma concentration required for these effects is unlikely to be reached by dietary intake. It must be stressed that these studies were conducted in models using cell lines that may not be representative of the disease.

Retinoids interfere in the DNA methylation mechanisms intervening in the one-carbon metabolism upregulating the glycine-N-methyltransferase and their cancer preventive activity has been extensively studied [158, 159]. Retinoids revert the cancer phenotype of breast and promyelocytic leukemia by demethylation and reactivation of the* RARβ2* receptor [160–162] and a recent epigenome-wide analysis identified a subset of genes, including stem cell genes, selectively modulated by retinoids [163].

Retinoids, polyphenols, and fatty acids can target the polycomb transcriptional repressor complexes that participate in the epigenetic silencing through methylation of Histone 3 (H3K27me3) [164]. Retinoic acid displaces PcG from its target genes including* HOXA1* and* RARβ2* [165], whereas ECGC reduces the expression of the PcG components* BMI1* and* EZH2*, slows the proliferation of cancer cells, and promotes apoptosis. A similar effect is exerted by curcumin [166, 167].

The inhibition of breast cancer cells growth was observed by dietary omega-3 fatty acids treatment and was related to the downmodulation of* EZH2* [168].

## 5. Conclusions

We have summarized the effects of environmental and of some lifestyle factors on epigenetic regulatory processes and we have reported the results of studies showing that some dietary components may have a role in cancer prevention.

Many environmental factors, including mother's diet and chemical pollution, can induce epigenetic alterations at conception or later in the uterine life. However, the phenotypic consequences of these early-life modifications should be still disclosed in full. Moreover, although epigenetic marks are considered inheritable, transgenerational epigenetic inheritance can be the result of confounding mechanisms such as cryptic genomic variations, behavioral or microbial effects.

Overall, many experimental evidences indicate that certain food components may interfere with cancer development and growth through epigenetic mechanisms. However, much work is needed to efficiently translate the promising results obtained* in vitro* and in animal models to the health system. In this respect, the antineoplastic properties of folates were not confirmed in clinical trials [148]. Moreover, it remains to be precisely established if the oral administration of unsupplemented food is sufficient to gain clinically relevant dosages of bioactive compounds, if there is a preferred timing of the life when the addition of food components could be effective and the extent and the duration of the biological effect. In conclusion, the now standardized technologies of epigenome analysis coupled with whole-genome expression studies are required to determine the biological and clinical impact of environmentally induced epigenetic modifications in humans.

## Figures and Tables

**Table 1:** FDA approved epigenetic drugs.

Common name	Trade name	Disease	Route	Mode of action
SAHA, vorinostat	Zolinza	CTCL	PO	HDAC inhibitor
Romidepsin	Istodax	CTCL	IV	HDAC inhibitor
5-Azacitidine	Vidaza	MDS	IV	DNMT inhibitor
5-Aza-2′-deoxycytidine	Dacogen	MDS	IV	DNMT inhibitor

CTCL: cutaneous T cell lymphoma.

MDS: myelodisplastic syndromes.

**Table 2:** Epigenetic modifier drugs in noncancer clinical trials (partial list).

Drug	Disease	NCT number	Phase
5-Azacitidine	Beta thalassemia	NCT00005934	Phase 2
5-Azacytidine + Na phenylbutyrate	Thalassemia major	NCT00007072	Phase 2
Resveratrol	Cardiovascular diseases	NCT01449110	Phase 2
Resveratrol	Trauma	NCT01321151	Phase 1
Resveratrol	Metabolic syndrome, obesity	NCT01150955	Phase 1
Curcumin	Irritable bowel syndrome	NCT00779493	Phase 4
Curcumin	Alzheimer disease	NCT00164749	Phase 2
Curcumin	Psoriasis	NCT00235625	Phase 2

**Table 3:** Enzymes involved in epigenetic modifications.

Enzyme	*N*	Function
DNA methyltransferase (DNMT)	5	
Histone Acethyltransferase (HAT)	19	Writer
Histone methyltransferase (HMT)	41	

Histone deacethylase (HDAC)	13	Eraser
Histone demethylase (KDM)	26	

Methyl binding proteins (MBD1)	5	
Proteins that recognize and react to specific modified histone residue	*N*	Readers

Total (April 2012)	>109	

**Table 4:** Effect of histone modifications on transcription in mammals.

Modification	Histone	Site	Effect
Acetylation	H2A	Lys5	Activation
	H2B	Lys5	Activation
		Lys12	Activation
		Lys15	Activation
		Lys20	Activation
	H3	Lys4	Activation
		Lys9	Activation
		Lys14	Activation
		Lys18	Activation
		Lys23	Activation
		Lys27	Activation
	H4	Lys5	Activation
		Lys8	Activation
		Lys12	Activation
		Lys16	Activation

Methylation	H1	Lys26	Repression
	H3	Lys4	Activation
		Arg8	Repression
		Lys9	Repression
		Arg17	Activation
		Lys27	Repression
		Lys36	Activation
		Lys79	Activation
	H4	Lys20	Repression
		Lys59	Repression

Phosphorylation	H1	Ser27	Activation
	H2A	Ser1	Repression
	H3	Ser10	Activation
		Ser28	Activation

Ubiquitylation	H2B	Lys120	Activation

**Table 5:** Principal ncRNAs.

ncRNA	Length (bp)	Function
miRNA	21–23	mRNA targeting
siRNA	20–25	Targeting of specific genes by sequence complementarity
piRNA	27–30	Chromatin regulation and transposon silencing
XiRNA	24–42	Control X chromosome methylation and inactivation
Long ncRNA	>200	Various, including targeting of specific genes

## List of Abbreviations

5-hmC: 5-Hydroxymethylcytosine
5-mC: 5-Methylcytosine
APP: Amyloid beta (A4) precursor protein
BPA: Bisphenol A
DES: Diethylstilbestrol
DNMT: DNA
DMPK: Dystrophia myotonica-protein kinase
EDC: Endocrine disrupting chemicals
EGCG: Epigallocatechin-3-gallate
HAT: Histone Acethyltransferase
ICF syndrome: Immunodeficiency, centromeric instability, and Facial dysmorphism syndrome
KDM: Lysine histone demethylase
MDB: Methyl DNA binding proteins
MLH1: mutL homolog 1
miRNA: Micro-RNA
ncRNA: Noncoding RNA
PcG: Polycomb proteins
RNAi: RNA interference
SAHA: Suberanilohydroxamic acid
SWI/SNF: SWItch/sucrose nonfermentable complex
TET 1: Ten-eleven translocation methylcytosine dioxygenase 1 enzymes
TRPM7: Transient receptor potential cation channel, member 7
TSA: Trichostatin A
UBE3A: Ubiquitin protein ligase E3A
Xist: X-inactive specific transcript (ncRNA).

## References

[1] Gosden R. G., Feinberg A. P. (2007). Genetics and epigenetics—nature's pen-and-pencil set. *The New England Journal of Medicine*.

[2] Rivera C. M., Ren B. (2013). Mapping human epigenomes. *Cell*.

[3] Choi J. D., Lee J. S. (2013). Interplay between epigenetics and genetics in cancer. *Genomics & Informatics*.

[4] Easwaran H., Tsai H.-C., Baylin S. B. (2014). Cancer epigenetics: tumor heterogeneity, plasticity of stem-like states, and drug resistance. *Molecular Cell*.

[5] Campbell R. M., Tummino P. J. (2014). Cancer epigenetics drug discovery and development: the challenge of hitting the mark. *The Journal of Clinical Investigation*.

[6] Kanherkar R. R., Bhatia-Dey N., Csoka A. B. (2014). Epigenetics across the human lifespan. *Frontiers in Cell and Developmental Biology*.

[7] Tammen S. A., Friso S., Choi S.-W. (2013). Epigenetics: the link between nature and nurture. *Molecular Aspects of Medicine*.

[8] Bernal A. J., Jirtle R. L. (2010). Epigenomic disruption: the effects of early developmental exposures. *Birth defects research Part A: Clinical and molecular teratology*.

[9] Rozek L. S., Dolinoy D. C., Sartor M. A., Omenn G. S. (2014). Epigenetics: relevance and implications for public health. *Annual Review of Public Health*.

[10] Ivanov M., Kacevska M., Ingelman-Sundberg M. (2012). Epigenomics and interindividual differences in drug response. *Clinical Pharmacology and Therapeutics*.

[11] Rius M., Lyko F. (2012). Epigenetic cancer therapy: rationales, targets and drugs. *Oncogene*.

[12] Sharma S. V., Lee D. Y., Li B. (2010). A chromatin-mediated reversible drug-tolerant state in cancer cell subpopulations. *Cell*.

[13] Glasspool R. M., Teodoridis J. M., Brown R. (2006). Epigenetics as a mechanism driving polygenic clinical drug resistance. *British Journal of Cancer*.

[14] Strik H. M., Buhk J.-H., Wrede A. (2008). Rechallenge with temozolomide with different scheduling is effective in recurrent malignant gliomas. *Molecular Medicine Reports*.

[15] Perry J. R., Rizek P., Cashman R., Morrison M., Morrison T. (2008). Temozolomide rechallenge in recurrent malignant glioma by using a continuous temozolomide schedule: the ‘rescue’ approach. *Cancer*.

[16] Chen X., Pan Y., Zhang S. (2014). Rechallenge with gefitinib following severe drug-induced hepatotoxicity in a patient with advanced non-small cell lung cancer: a case report and literature review. *Oncology Letters*.

[17] Gasparotto D., Miolo G., Torrisi E. (2014). Improved outcome with multimodal treatment and imatinib rechallenge in advanced GIST. *International Journal of Colorectal Disease*.

[18] Matei D., Fang F., Shen C. (2012). Epigenetic resensitization to platinum in ovarian cancer. *Cancer Research*.

[19] Riggs A. D., Jones P. A. (1983). 5-methylcytosine, gene regulation, and cancer. *Advances in Cancer Research*.

[20] Xu F., Mao C., Ding Y. (2010). Molecular and enzymatic profiles of mammalian DNA methyltransferases: structures and targets for drugs. *Current Medicinal Chemistry*.

[21] Nile C. J., Read R. C., Akil M., Duff G. W., Wilson A. G. (2008). Methylation status of a single CpG site in the IL6 promoter is related to IL6 messenger RNA levels and rheumatoid arthritis. *Arthritis and Rheumatism*.

[22] Pogribny I. P., Pogribna M., Christman J. K., James S. J. (2000). Single-site methylation within the p53 promoter region reduces gene expression in a reporter gene construct: possible *in vivo* relevance during tumorigenesis. *Cancer Research*.

[23] Herman J. G., Baylin S. B. (2003). Gene silencing in cancer in association with promoter hypermethylation. *The New England Journal of Medicine*.

[24] Deaton A. M., Bird A. (2011). CpG islands and the regulation of transcription. *Genes and Development*.

[25] Casciano I., Banelli B., Croce M. (2002). Role of methylation in the control of Δ*Np73* expression in neuroblastoma. *Cell Death and Differentiation*.

[26] Casciano I., Mazzocco K., Boni L. (2002). Expression of ΔNp73 is a molecular marker for adverse outcome in neuroblastoma patients. *Cell Death and Differentiation*.

[27] Romani M., Tonini G. P., Banelli B. (2003). Biological and clinical role of p73 in neuroblastoma. *Cancer Letters*.

[28] Smith J. F., Mahmood S., Song F. (2007). Identification of DNA methylation in 3′ genomic regions that are associated with upregulation of gene expression in colorectal cancer. *Epigenetics*.

[29] Weber M., Davies J. J., Wittig D. (2005). Chromosome-wide and promoter-specific analyses identify sites of differential DNA methylation in normal and transformed human cells. *Nature Genetics*.

[30] Azzi S., Habib W. A., Netchine I. (2014). Beckwith-Wiedemann and Russell-Silver Syndromes: from new molecular insights to the comprehension of imprinting regulation. *Current Opinion in Endocrinology, Diabetes and Obesity*.

[31] Walton E., Francastel C., Velasco G. (2014). Dnmt3b prefers germ line genes and centromeric regions: lessons from the ICF syndrome and cancer and implications for diseases. *Biology*.

[32] Rangasamy S., D'Mello S. R., Narayanan V. (2013). Epigenetics, autism spectrum, and neurodevelopmental disorders. *Neurotherapeutics*.

[33] Kraus T. F., Guibourt V., Kretzschmar H. A. (2014). 5-hydroxymethylcytosine, the ‘sixth base’, during brain development and ageing. *Journal of Neural Transmission*.

[34] Wang J., Tang J., Lai M., Zhang H. (2014). 5-Hydroxymethylcytosine and disease. *Mutation Research/Reviews in Mutation Research*.

[35] Allfrey V. G., Mirsky A. E. (1964). Structural modifications of histones and their possible role in the regulation of RNA synthesis. *Science*.

[36] Vidali G., Gershey E. L., Allfrey V. G. (1968). Chemical studies of histone acetylation. The distribution of epsilon-N-acetyllysine in calf thymus histones. *The Journal of Biological Chemistry*.

[37] Polo S. E. (2014). Reshaping chromatin after DNA damage: the choreography of histone proteins. *Journal of Molecular Biology*.

[38] Weber C. M., Henikoff S. (2014). Histone variants: dynamic punctuation in transcription. *Genes & Development*.

[39] Jenuwein T., Allis C. D. (2001). Translating the histone code. *Science*.

[40] Allfrey V. G., Faulkner R., Mirsky A. E. (1964). Acetylation and methylation of histones and their possible role in the regulation of Rna synthesis. *Proceedings of the National Academy of Sciences of the United States*.

[41] Shi Y., Lan F., Matson C. (2004). Histone demethylation mediated by the nuclear amine oxidase homolog LSD1. *Cell*.

[42] Pasini D., Bracken A. P., Agger K. (2008). Regulation of stem cell differentiation by histone methyltransferases and demethylases. *Cold Spring Harbor Symposia on Quantitative Biology*.

[43] Cloos P. A. C., Christensen J., Agger K., Helin K. (2008). Erasing the methyl mark: histone demethylases at the center of cellular differentiation and disease. *Genes and Development*.

[44] Agger K., Christensen J., Cloos P. A., Helin K. (2008). The emerging functions of histone demethylases. *Current Opinion in Genetics and Development*.

[45] Helin K., Dhanak D. (2013). Chromatin proteins and modifications as drug targets. *Nature*.

[46] Rotili D., Mai A. (2011). Targeting histone demethylases: a new avenue for the fight against cancer. *Genes and Cancer*.

[47] Havas K., Flaus A., Phelan M. (2000). Generation of superhelical torsion by ATP-dependent chromatin remodeling activities. *Cell*.

[48] Dechassa M. L., Sabri A., Pondugula S. (2010). SWI/SNF has intrinsic nucleosome disassembly activity that is dependent on adjacent nucleosomes. *Molecular Cell*.

[49] Kwaks T. H., Barnett P., Hemrika W. (2003). Identification of anti-repressor elements that confer high and stable protein production in mammalian cells. *Nature Biotechnology*.

[50] Zhou W., Zhu P., Wang J. (2008). Histone H2A monoubiquitination represses transcription by inhibiting RNA polymerase II transcriptional elongation. *Molecular Cell*.

[51] Kanhere A., Viiri K., Araújo C. C. (2010). Short RNAs are transcribed from repressed polycomb target genes and interact with polycomb repressive complex-2. *Molecular Cell*.

[52] Zhao J., Sun B. K., Erwin J. A., Song J.-J., Lee J. T. (2008). Polycomb proteins targeted by a short repeat RNA to the mouse X chromosome. *Science*.

[53] Ranganathan K., Sivasankar V. (2014). MicroRNAs—Biology and clinical applications. *Journal of Oral and Maxillofacial Pathology*.

[54] Bartel D. P. (2004). MicroRNAs: genomics, biogenesis, mechanism, and function. *Cell*.

[55] He L., Hannon G. J. (2004). MicroRNAs: small RNAs with a big role in gene regulation. *Nature Reviews Genetics*.

[56] Calin G. A., Croce C. M. (2006). MicroRNA signatures in human cancers. *Nature Reviews Cancer*.

[57] Barski A., Jothi R., Cuddapah S. (2009). Chromatin poises miRNA- and protein-coding genes for expression. *Genome Research*.

[58] Sato F., Tsuchiya S., Meltzer S. J., Shimizu K. (2011). MicroRNAs and epigenetics. *FEBS Journal*.

[59] Fabbri M., Garzon R., Cimmino A. (2007). MicroRNA-29 family reverts aberrant methylation in lung cancer by targeting DNA methyltransferases 3A and 3B. *Proceedings of the National Academy of Sciences of the United States of America*.

[60] Zhang L., Hou D., Chen X. (2012). Exogenous plant MIR168a specifically targets mammalian LDLRAP1: evidence of cross-kingdom regulation by microRNA. *Cell Research*.

[61] Richardson B. (2007). Primer: epigenetics of autoimmunity. *Nature Clinical Practice Rheumatology*.

[62] Bollati V., Baccarelli A. (2010). Environmental epigenetics. *Heredity*.

[63] Baccarelli A., Bollati V. (2009). Epigenetics and environmental chemicals. *Current Opinion in Pediatrics*.

[64] Gluckman P. D., Hanson M. A., Buklijas T. (2010). A conceptual framework for the developmental origins of health and disease. *Journal of Developmental Origins of Health and Disease*.

[65] Hanson M. A., Gluckman P. D. (2008). Developmental origins of health and disease: new insights. *Basic & Clinical Pharmacology & Toxicology*.

[66] Gorelik G. J., Yarlagadda S., Richardson B. C. (2012). Protein kinase C*δ* oxidation contributes to ERK inactivation in lupus T cells. *Arthritis and Rheumatism*.

[67] Tommasi S., Kim S.-I., Zhong X., Wu X., Pfeifer G. P., Besaratinia A. (2010). Investigating the epigenetic effects of a prototype smoke-derived carcinogen in human cells. *PLoS ONE*.

[68] Marwick J. A., Kirkham P. A., Stevenson C. S. (2004). Cigarette smoke alters chromatin remodeling and induces proinflammatory genes in rat lungs. *American Journal of Respiratory Cell and Molecular Biology*.

[69] Liu F., Killian J. K., Yang M. (2010). Epigenomic alterations and gene expression profiles in respiratory epithelia exposed to cigarette smoke condensate. *Oncogene*.

[70] Boyle P., Autier P., Bartelink H. (2003). European code against cancer and scientific justification: third version (2003). *Annals of Oncology*.

[71] Van Engeland M., Weijenberg M. P., Roemen G. M. J. M. (2003). Effects of dietary folate and alcohol intake on promoter methylation in sporadic colorectal cancer: the Netherlands cohort study on diet and cancer. *Cancer Research*.

[72] de Vogel S., Bongaerts B. W. C., Wouters K. A. D. (2008). Associations of dietary methyl donor intake with *MLH1* promoter hypermethylation and related molecular phenotypes in sporadic colorectal cancer. *Carcinogenesis*.

[73] Lehmann U., Hasemeier B., Lilischkis R., Kreipe H. (2001). Quantitative analysis of promoter hypermethylation in laser-microdissected archival specimens. *Laboratory Investigation*.

[74] Banelli B., Bonassi S., Casciano I. (2010). Outcome prediction and risk assessment by quantitative pyrosequencing methylation analysis of the SFN gene in advanced stage, high-risk, neuroblastic tumor patients. *International Journal of Cancer*.

[75] Vinci A. D., Casciano I., Marasco E. (2012). Quantitative methylation analysis of *HOXA3, 7, 9*, and *10* genes in glioma: association with tumor WHO grade and clinical outcome. *Journal of Cancer Research and Clinical Oncology*.

[76] Di Vinci A., Brigati C., Casciano I. (2012). HOXA7, 9, and 10 are methylation targets associated with aggressive behavior in meningiomas. *Translational Research*.

[77] Banelli B., Merlo D. F., Allemanni G., Forlani A., Romani M. (2013). Clinical potentials of methylator phenotype in stage 4 high-risk neuroblastoma: an open challenge. *PLoS ONE*.

[78] Brakensiek K., Wingen L. U., Länger F., Kreipe H., Lehmann U. (2007). Quantitative high-resolution CpG island mapping with pyrosequencing reveals disease-specific methylation patterns of the CDKN2B gene in myelodysplastic syndrome and myeloid leukemia. *Clinical Chemistry*.

[79] Liu Y., Balaraman Y., Wang G., Nephew K. P., Zhou F. C. (2009). Alcohol exposure alters DNA methylation profiles in mouse embryos at early neurulation. *Epigenetics*.

[80] Diamanti-Kandarakis E., Bourguignon J.-P., Giudice L. C. (2009). Endocrine-disrupting chemicals: an Endocrine Society scientific statement. *Endocrine Reviews*.

[81] Anway M. D., Skinner M. K. (2006). Epigenetic transgenerational actions of endocrine disruptors. *Endocrinology*.

[82] Anway M. D., Cupp A. S., Uzumcu N., Skinner M. K. (2005). Toxicology: epigenetic transgenerational actions of endocrine disruptors and male fertility. *Science*.

[83] Skinner M. K., Manikkam M., Tracey R., Guerrero-Bosagna C., Haque M., Nilsson E. E. (2013). Ancestral dichlorodiphenyltrichloroethane (DDT) exposure promotes epigenetic transgenerational inheritance of obesity. *BMC Medicine*.

[84] Tracey R., Manikkam M., Guerrero-Bosagna C., Skinner M. K. (2013). Hydrocarbons (jet fuel JP-8) induce epigenetic transgenerational inheritance of obesity, reproductive disease and sperm epimutations. *Reproductive Toxicology*.

[85] Skinner M. K. (2014). Environmental stress and epigenetic transgenerational inheritance. *BMC Medicine*.

[86] Nilsson E. E., Skinner M. K. (2014). Environmentally induced epigenetic transgenerational inheritance of disease susceptibility. *Translational Research*.

[87] Grossniklaus U., Kelly B., Ferguson-Smith A. C., Pembrey M., Lindquist S. (2013). Transgenerational epigenetic inheritance: how important is it?. *Nature Reviews Genetics*.

[88] Heard E., Martienssen R. A. (2014). Transgenerational epigenetic inheritance: myths and mechanisms. *Cell*.

[89] Doherty L. F., Bromer J. G., Zhou Y., Aldad T. S., Taylor H. S. (2010). In utero exposure to diethylstilbestrol (DES) or bisphenol-A (BPA) increases EZH2 expression in the mammary gland: an epigenetic mechanism linking endocrine disruptors to breast cancer. *Hormones and Cancer*.

[90] Veiga-Lopez A., Luense L. J., Christenson L. K., Padmanabhan V. (2013). Developmental programming: gestational bisphenol-A treatment alters trajectory of fetal ovarian gene expression. *Endocrinology*.

[91] Ho S.-M., Tang W.-Y., Belmonte de Frausto J., Prins G. S. (2006). Developmental exposure to estradiol and bisphenol A increases susceptibility to prostate carcinogenesis and epigenetically regulates phosphodiesterase type 4 variant 4. *Cancer Research*.

[92] Dolinoy D. C., Huang D., Jirtle R. L. (2007). Maternal nutrient supplementation counteracts bisphenol A-induced DNA hypomethylation in early development. *Proceedings of the National Academy of Sciences of the United States of America*.

[93] Robboy S. J., Scully R. E., Welch W. R., Herbst A. L. (1977). Intrauterine diethylstilbestrol exposure and its consequences: pathologic characteristics of vaginal adenosis, clear cell adenocarcinoma, and related lesions. *Archives of Pathology and Laboratory Medicine*.

[94] Li S., Washburn K. A., Moore R. (1997). Developmental exposure to diethylstilbestrol elicits demethylation of estrogen-responsive lactoferrin gene in mouse uterus. *Cancer Research*.

[95] Skinner M. K. (2011). Environmental epigenetic transgenerational inheritance and somatic epigenetic mitotic stability. *Epigenetics*.

[96] Karsli-Ceppioglu S., Dagdemir A., Judes G. (2014). Epigenetic mechanisms of breast cancer: an update of the current knowledge. *Epigenomics*.

[97] Zhang K., Sun X., Zhou X. (2015). Long non-coding RNA HOTAIR promotes glioblastoma cell cycle progression in an EZH2 dependent manner. *Oncotarget*.

[98] Murat A., Migliavacca E., Gorlia T. (2008). Stem cell-related ‘self-renewal’ signature and high epidermal growth factor receptor expression associated with resistance to concomitant chemoradiotherapy in glioblastoma. *Journal of Clinical Oncology*.

[99] Zama A. M., Uzumcu M. (2009). Fetal and neonatal exposure to the endocrine disruptor methoxychlor causes epigenetic alterations in adult ovarian genes. *Endocrinology*.

[100] Stouder C., Paoloni-Giacobino A. (2011). Specific transgenerational imprinting effects of the endocrine disruptor methoxychlor on male gametes. *Reproduction*.

[101] Stouder C., Paoloni-Giacobino A. (2010). Transgenerational effects of the endocrine disruptor vinclozolin on the methylation pattern of imprinted genes in the mouse sperm. *Reproduction*.

[102] Armenti A. E., Zama A. M., Passantino L., Uzumcu M. (2008). Developmental methoxychlor exposure affects multiple reproductive parameters and ovarian folliculogenesis and gene expression in adult rats. *Toxicology and Applied Pharmacology*.

[103] Bartsch H., Nair J. (2004). Oxidative stress and lipid peroxidation-derived DNA-lesions in inflammation driven carcinogenesis. *Cancer Detection and Prevention*.

[104] Lawless M. W., O'Byrne K. J., Gray S. G. (2009). Oxidative stress induced lung cancer and COPD: opportunities for epigenetic therapy. *Journal of Cellular and Molecular Medicine*.

[105] Druesne N., Pagniez A., Mayeur C. (2004). Diallyl disulfide (DADS) increases histone acetylation and p21^waf1/cip1^ expression in human colon tumor cell lines. *Carcinogenesis*.

[106] Kiec-Wilk B., Razny U., Mathers J. C., Dembinska-Kiec A. (2009). DNA methylation, induced by beta-carotene and arachidonic acid, plays a regulatory role in the pro-angiogenic VEGF-receptor (KDR) gene expression in endothelial cells. *Journal of Physiology and Pharmacology*.

[107] Chen J., Xu X. (2010). Diet, epigenetic, and cancer prevention. *Advances in Genetics*.

[108] Dashwood R. H., Ho E. (2007). Dietary histone deacetylase inhibitors: from cells to mice to man. *Seminars in Cancer Biology*.

[109] Mckay J. A., Mathers J. C. (2011). Diet induced epigenetic changes and their implications for health. *Acta Physiologica*.

[110] Cravo M., Fidalgo P., Pereira A. D. (1994). DNA methylation as an intermediate biomarker in colorectal cancer: modulation by folic acid supplementation. *European Journal of Cancer Prevention*.

[111] Kim Y.-I., Baik H. W., Fawaz K. (2001). Effects of folate supplementation on two provisional molecular markers of colon cancer: a prospective, randomized trial. *American Journal of Gastroenterology*.

[112] Pufulete M., Al-Ghnaniem R., Khushal A. (2005). Effect of folic acid supplementation on genomic DNA methylation in patients with colorectal adenoma. *Gut*.

[113] Rampersaud G. C., Kauwell G. P. A., Hutson A. D., Cerda J. J., Bailey L. B. (2000). Genomic DNA methylation decreases in response to moderate folate depletion in elderly women. *American Journal of Clinical Nutrition*.

[114] Steegers-Theunissen R. P., Obermann-Borst S. A., Kremer D. (2009). Periconceptional maternal folic acid use of 400 *μ*g per day is related to increased methylation of the *IGF2* gene in the very young child. *PLoS ONE*.

[115] Sakatani T., Kaneda A., Iacobuzio-Donahue C. A. (2005). Loss of imprinting of Igf2 alters intestinal maturation and tumorigenesis in mice. *Science*.

[116] Davison J. M., Mellott T. J., Kovacheva V. P., Blusztajn J. K. (2009). Gestational choline supply regulates methylation of histone H3, expression of histone methyltransferases G9a (Kmt1c) and Suv39h1 (Kmt1a), and DNA methylation of their genes in rat fetal liver and brain. *The Journal of Biological Chemistry*.

[117] Lumey L. H., Stein A. D., Kahn H. S. (2007). Cohort profile: the Dutch Hunger Winter families study. *International Journal of Epidemiology*.

[118] Heijmans B. T., Tobi E. W., Stein A. D. (2008). Persistent epigenetic differences associated with prenatal exposure to famine in humans. *Proceedings of the National Academy of Sciences of the United States of America*.

[119] Fini L., Selgrad M., Fogliano V. (2007). Annurca apple polyphenols have potent demethylating activity and can reactivate silenced tumor suppressor genes in colorectal cancer cells. *Journal of Nutrition*.

[120] Link A., Balaguer F., Goel A. (2010). Cancer chemoprevention by dietary polyphenols: promising role for epigenetics. *Biochemical Pharmacology*.

[121] Paluszczak J., Krajka-Kuźniak V., Małecka Z. (2011). Frequent gene hypermethylation in laryngeal cancer cell lines and the resistance to demethylation induction by plant polyphenols. *Toxicology in Vitro*.

[122] Fang M. Z., Wang Y., Ai N. (2003). Tea polyphenol (-)-epigallocatechin-3-gallate inhibits DNA methyltransferase and reactivates methylation-silenced genes in cancer cell lines. *Cancer Research*.

[123] Lee W. J., Shim J.-Y., Zhu B. T. (2005). Mechanisms for the inhibition of DNA methyltransferases by tea catechins and bioflavonoids. *Molecular Pharmacology*.

[124] Yuasa Y., Nagasaki H., Akiyama Y. (2009). DNA methylation status is inversely correlated with green tea intake and physical activity in gastric cancer patients. *International Journal of Cancer*.

[125] Yuasa Y., Nagasaki H., Akiyama Y. (2005). Relationship between *CDX2* gene methylation and dietary factors in gastric cancer patients. *Carcinogenesis*.

[126] Qin W., Zhu W., Shi H. (2009). Soy isoflavones have an antiestrogenic effect and alter mammary promoter hypermethylation in healthy premenopausal women. *Nutrition and Cancer*.

[127] Li Y., Liu L., Andrews L. G., Tollefsbol T. O. (2009). Genistein depletes telomerase activity through cross-talk between genetic and epigenetic mechanisms. *International Journal of Cancer*.

[128] Cross H. S., Kállay E., Lechner D., Gerdenitsch W., Adlercreutz H., Armbrecht H. J. (2004). Phytoestrogens and vitamin D metabolism: a new concept for the prevention and therapy of colorectal, prostate, and mammary carcinomas. *Journal of Nutrition*.

[129] Fiala E. S., Staretz M. E., Pandya G. A., El-Bayoumy K., Hamilton S. R. (1998). Inhibition of DNA cytosine methyltransferase by chemopreventive selenium compounds, determined by an improved assay for DNA cytosine methyltransferase and DNA cytosine methylation. *Carcinogenesis*.

[130] Xiang N., Zhao R., Song G., Zhong W. (2008). Selenite reactivates silenced genes by modifying DNA methylation and histones in prostate cancer cells. *Carcinogenesis*.

[131] McGowan P. O., Sasaki A., D'Alessio A. C. (2009). Epigenetic regulation of the glucocorticoid receptor in human brain associates with childhood abuse. *Nature Neuroscience*.

[132] Tsankova N. M., Berton O., Renthal W., Kumar A., Neve R. L., Nestler E. J. (2006). Sustained hippocampal chromatin regulation in a mouse model of depression and antidepressant action. *Nature Neuroscience*.

[133] Issa J.-P. J. (2007). DNA methylation as a therapeutic target in cancer. *Clinical Cancer Research*.

[134] Tost J. (2010). DNA methylation: an introduction to the biology and the disease-associated changes of a promising biomarker. *Molecular Biotechnology*.

[135] McCabe M. T., Brandes J. C., Vertino P. M. (2009). Cancer DNA methylation: molecular mechanisms and clinical implications. *Clinical Cancer Research*.

[136] Eden A., Gaudet F., Waghmare A., Jaenisch R. (2003). Chromosomal instability and tumors promoted by DNA hypomethylation. *Science*.

[137] Weber B., Kimhi S., Howard G., Eden A., Lyko F. (2010). Demethylation of a LINE-1 antisense promoter in the cMet locus impairs Met signalling through induction of illegitimate transcription. *Oncogene*.

[138] Wolff E. M., Byun H.-M., Han H. F. (2010). Hypomethylation of a LINE-1 promoter activates an alternate transcript of the MET oncogene in bladders with cancer. *PLoS Genetics*.

[139] Lizardi P. M. (2010). As we bring demethylating drugs to the clinic, we better know the DICE being cast. *Oncogene*.

[140] Hansen K. D., Timp W., Bravo H. C. (2011). Increased methylation variation in epigenetic domains across cancer types. *Nature Genetics*.

[141] Issa J.-P. J., Garcia-Manero G., Giles F. J. (2004). Phase 1 study of low-dose prolonged exposure schedules of the hypomethylating agent 5-aza-2′-deoxycytidine (decitabine) in hematopoietic malignancies. *Blood*.

[142] Kaminskas E., Farrell A., Abraham S. (2005). Approval summary: azacitidine for treatment of myelodysplastic syndrome subtypes. *Clinical Cancer Research*.

[143] Issa J.-P. F., Gharibyan V., Cortes J. (2005). Phase II study of low-dose decitabine in patients with chronic myelogenous leukemia resistant to imatinib mesylate. *Journal of Clinical Oncology*.

[144] Cameron E. E., Bachman K. E., Myöhänen S., Herman J. G., Baylin S. B. (1999). Synergy of demethylation and histone deacetylase inhibition in the re-expression of genes silenced in cancer. *Nature Genetics*.

[145] Sie K. K. Y., Medline A., Van Weel J. (2011). Effect of maternal and postweaning folic acid supplementation on colorectal cancer risk in the offspring. *Gut*.

[146] Ly A., Lee H., Chen J. (2011). Effect of maternal and postweaning folic acid supplementation on mammary tumor risk in the offspring. *Cancer Research*.

[147] Keyes M. K., Jang H., Mason J. B. (2007). Older age and dietary folate are determinants of genomic and p16-specific DNA methylation in mouse colon. *Journal of Nutrition*.

[148] Cole B. F., Baron J. A., Sandler R. S. (2007). Folic acid for the prevention of colorectal adenomas: a randomized clinical trial. *Journal of the American Medical Association*.

[149] Wallace K., Grau M. V., Levine A. J. (2010). Association between folate levels and CpG island hypermethylation in normal colorectal mucosa. *Cancer Prevention Research*.

[150] Fang M., Chen D., Yang C. S. (2007). Dietary polyphenols may affect DNA methylation. *Journal of Nutrition*.

[151] Berletch J. B., Liu C., Love W. K., Andrews L. G., Katiyar S. K., Tollefsbol T. O. (2008). Epigenetic and genetic mechanisms contribute to telomerase inhibition by EGCG. *Journal of Cellular Biochemistry*.

[152] Chuang J. C., Yoo C. B., Kwan J. M. (2005). Comparison of biological effects of non-nucleoside DNA methylation inhibitors versus 5-aza-2′-deoxycytidine. *Molecular Cancer Therapeutics*.

[153] Taylor C. K., Levy R. M., Elliott J. C., Burnett B. P. (2009). The effect of genistein aglycone on cancer and cancer risk: a review of *in vitro*, preclinical, and clinical studies. *Nutrition Reviews*.

[154] Fang M. Z., Chen D., Sun Y., Jin Z., Christman J. K., Yang C. S. (2005). Reversal of hypermethylation and reactivation of p16INK4a, RAR*β*, and MGMT genes by genistein and other isoflavones from soy. *Clinical Cancer Research*.

[155] Majid S., Dar A. A., Ahmad A. E. (2009). BTG3 tumor suppressor gene promoter demethylation, histone modification and cell cycle arrest by genistein in renal cancer. *Carcinogenesis*.

[156] Day J. K., Bauer A. M., Desbordes C. (2002). Genistein alters methylation patterns in mice. *Journal of Nutrition*.

[157] Constantinou A. I., Lantvit D., Hawthorne M., Xu X., van Breemen R. B., Pezzuto J. M. (2001). Chemopreventive effects of soy protein and purified soy isoflavones on DMBA-induced mammary tumors in female Sprague-Dawley rats. *Nutrition and Cancer*.

[158] Johanning G. L., Piyathilake C. J. (2003). Retinoids and epigenetic silencing in cancer. *Nutrition Reviews*.

[159] Rowling M. J., McMullen M. H., Schalinske K. L. (2002). Vitamin A and its derivatives induce hepatic glycine N-methyltransferase and hypomethylation of DNA in rats. *Journal of Nutrition*.

[160] Esteller M., Guo M., Moreno V. (2002). Hypermethylation-associated inactivation of the cellular retinol-binding-protein 1 gene in human cancer. *Cancer Research*.

[161] Di Croce L., Raker V. A., Corsaro M. (2002). Methyltransferase recruitment and DNA hypermethylation of target promoters by an oncogenic transcription factor. *Science*.

[162] Fazi F., Travaglini L., Carotti D. (2005). Retinoic acid targets DNA-methyltransferases and histone deacetylases during APL blast differentiation *in vitro* and *in vivo*. *Oncogene*.

[163] Cheong H. S., Lee H. C., Park B. L. (2010). Epigenetic modification of retinoic acid-treated human embryonic stem cells. *BMB Reports*.

[164] Bracken A. P., Helin K. (2009). Polycomb group proteins: navigators of lineage pathways led astray in cancer. *Nature Reviews Cancer*.

[165] Gudas L. J., Wagner J. A. (2011). Retinoids regulate stem cell differentiation. *Journal of Cellular Physiology*.

[166] Balasubramanian S., Adhikary G., Eckert R. L. (2010). The Bmi-1 polycomb protein antagonizes the (-)-epigallocatechin-3-gallate-dependent suppression of skin cancer cell survival. *Carcinogenesis*.

[167] Hua W.-F., Fu Y.-S., Liao Y.-J. (2010). Curcumin induces down-regulation of EZH2 expression through the MAPK pathway in MDA-MB-435 human breast cancer cells. *European Journal of Pharmacology*.

[168] Dimri M., Bommi P. V., Sahasrabuddhe A. A., Khandekar J. D., Dimri G. P. (2010). Dietary omega-3 polyunsaturated fatty acids suppress expression of EZH2 in breast cancer cells. *Carcinogenesis*.

